# High task demand in dual-target paradigm redirects experimentally increased anxiety to uphold goal-directed attention

**DOI:** 10.1177/03010066241232593

**Published:** 2024-03-04

**Authors:** Miloš Stanković, Fredrik Allenmark, Zhuanghua Shi

**Affiliations:** 9147 University of Regensburg, Germany; Ludwig-Maximilians-Universität München, Munich, Germany; Ludwig-Maximilians-Universität München, Germany; Ludwig-Maximilians-Universität München, Germany

**Keywords:** anxiety, attentional control, goal-directed attention, distractor, visual search

## Abstract

Previous research has shown that state anxiety facilitates stimulus-driven attentional capture and impairs goal-directed attentional control by increasing sensitivity to salient distractors or threat cues or narrowing spatial attention. However, recent findings in this area have been mixed, and less is known about how state-dependent anxiety may affect attentional performance. Here, we employed a novel dual-target search paradigm to investigate this relationship. This paradigm allowed us to investigate attentional control and how focus narrows under different anxiety states. Participants watched a short movie—either anxiety-inducing or neutral—before engaging in the dual-target visual search task. We found that they performed faster and more accurately in trials without the salient distractor compared to those with distractors, and they performed better in tasks presented on the center than the periphery. However, despite a significant increase in self-reported anxiety in the anxiety-inducing session, participants’ performance in terms of speed and accuracy remain comparable across both anxious and neutral sessions. This resilience is likely due to compensatory mechanisms that offset anxiety, a result of the high demands and working memory load inherent in the dual-target task.

In our study, we explored the intricate relationship between state anxiety and goal-directed attention using a unique dual-target visual search paradigm. Intriguingly, we found that increased state anxiety did not interfere with attentional capture and the central preference. We suggest that this resilience is likely due to compensatory mechanisms that offset anxiety.

For biological survival, our attention can be automatically shifted toward potential dangers, including social (e.g., an angry face) and physical threats. The presence of threat-related cues often triggers a rapid fear response (Ghassemzadeh et al., 2019). Fear also causes an immediate reaction to both conditioned or unconditioned stimuli (Ghassemzadeh et al., 2019), directing our attention toward a threat (Öhman et al., 2001; Vuilleumier, 2005). In contrast, anxiety elicits a slower response to uncertain impending threats (LeDoux & Pine, 2016). Goal-directed attention is a voluntary process, selecting and focusing on objects, locations, or features relevant to our current behavioral goals (Katsuki & Constantinidis, 2014), whereas stimulus-driven visual attention is an involuntary, automatic process that often leads to “attentional capture” when encountering a salient object (Neumann, 1984; Theeuwes & Failing, 2020).

It remains not fully understood regarding the impact of anxiety on goal-directed attentional control, whether it hinders or enhances it. Some theoretical accounts, such as attentional control theory (ACT), hold that anxiety leads to a focus on threatening stimuli due to a reduction in goal-directed attentional control (Eysenck & Derakshan, 2011) and an increase of distractibility (stimulus-driven attention) (Eysenck et al., 2007; Moser et al., 2012). In line with ACT, Moser et al. (2012) found that trait anxiety correlates with increased attentional distraction by color singleton in an additional singleton task. In contrast, Kim et al. (2021) found that mild threats might actually improve goal-directed attentional control, as shown in their electric shock conditioning study, which revealed more efficient visual search and fewer missed targets under threat. Yet, the specific circumstances under which anxiety modulates goal-directed attentional control, potentially impairing or enhancing behavioral performance, remain unclear.

Additionally, anxiety can be categorized both physiologically and psychometrically into worry/apprehension and arousal/emotionality dimensions. Worry involves verbal rumination about possible negative future outcomes, while arousal encompasses physiological hyperarousal and somatic tension (e.g., dizziness, high heart rate, sweaty palms, hypervigilance, etc.) occurring in panic (Moran, 2016). Some theories propose that worry, as opposed to arousal, is more amenable to down-regulation (Kalisch et al., 2006). This is based on the idea that arousal primes the mind to deal with potential threats (Cornwell et al., 2011). It implies that strategies targeting down-regulating worry might be more effective or accessible compared to down-regulating arousal, which might serve an adaptive function in preparing individuals for perceived threat.

Over the past three decades, research in visual attention has predominantly focused on goal-directed, stimulus-driven, and history-driven attention (Awh et al., 2012; Krummenacher et al., 2010; Theeuwes & Failing, 2020), although some studies have explored other types such as global and local attention (Allenmark et al., 2022; Stanković & Nešić, 2018). The additional singleton search paradigms (Allenmark et al., 2019; Goschy et al., 2014; Theeuwes, 1992), where observers search for a task-relevant unique shape (a shape singleton) amidst a task-irrelevant but physically salient distractor (e.g., a color singleton), is often a standard method for investigating stimulus-driven versus goal-directed attentional capture. Typically, this paradigm reveals that the singleton distractor cannot be fully suppressed (Theeuwes, 1992), causing attentional capture (i.e., distractor-interference) indicated by a higher error rate and/or prolonged latency of RT (but see Geyer et al., 2008). Schmidt et al. (2015) expanded on this by introducing fear-conditioning to the paradigm, associating an irrelevant non-target distractor (e.g., an orange or a blue diamond) with aversive stimuli (e.g., electric shocks), resulting in heightened attentional capture towards the fear-associated stimuli. Their paradigm, in which searching for a target is coupled with an aversive stimulus, differs from research in which searching for a neutral stimulus is performed under fear or anxiety (LeDoux & Pine, 2016). The internal mental state, ranging from neutral to fearful, interacts with the external world, spanning from neutral to aversive stimuli. Thus, prior research has provided inconsistent evidence in various attentional tasks regarding whether threat-induced anxiety may impair or facilitate visual search for neutral stimuli. For example, studies have shown both improved Stroop task performance (Hu et al., 2012) or impaired Stroop task performance (Choi et al., 2012) under the threat of electric shock, or an anti-saccade task (Cornwell et al., 2012) in which threat-induced anxiety impaired responding for an anti-saccades task but improved stimulus-driven responses (pro-saccades). The antisaccade paradigm (Hallett, 1978), where participants make eye movements (saccades) either toward or away from an suddenly appearing stimulus, has been often used in studies examining anxiety and attention relationships (Basanovic et al., 2023).

Additionally, attentional capture by an irrelevant, but salient distractor, can be less prominent when attention is narrowed to a small spatial region, such as using rapid stream visual presentation paradigm (Belopolsky & Theeuwes, 2010). In contrast, when spatial attention is broader, items across the visual field are processed in parallel. This leads to attention capture by salient items, independent of visual search goals (Belopolsky & Theeuwes, 2010). Visual search stimuli and their sizes play a significant role in attention (Stanković & Nešić, 2018). Critically, anxiety effect on attentional control has been linked to a spatial narrowing of attention (Derryberry & Reed, 1998; Najmi et al., 2012), focusing on fewer items and more towards the center than the periphery under intense anxiety (Janelle et al., 1992). For example, driving a car in an anxious state may lead to slower and less accurate evaluation and processing of task-relevant information in the peripheral visual field (Janelle et al., 1992). High anxiety trait contributes to individual differences in vulnerability to stress response (Weger & Sandi, 2018). Whereas the attentional window is a perceptual phenomenon, attentional narrowing typically refers to attention affected by emotional components. Early theories suggest that anxiety may narrow spatial attention (Easterbrook, 1959), shifting focus of attention from peripheral areas to central viewpoints, thus enhancing the processing of central information. As such, high anxiety could therefore affect the deployment of attention toward central points. However, this narrowing may limit the ability to attend to a broader region, causing potential higher error rates in peripheral tasks.

Recent studies, however, indicate that anxiety may differentially narrow observers’ attention. For example, the literature has demonstrated that the threat of electric shock may improve performance in the Stroop task (Robinson et al., 2013) or in a color-dot-detection task (Sussman et al., 2013), in which participants had to identify the color of a dot on the screen while negative pictures were presented on the background as aversive stimuli. Interestingly, Rogé et al. (2003) examined non-anxious participants in a monotonous driving task and reported that lower alertness, or low anxiety, led to narrowed spatial attention and degraded ability to detect peripheral versus central stimuli. These studies suggest that attentional narrowing, by reducing peripheral distractions and focusing on the central region, may enhance performance for the central at the cost of the peripheral. However, it remains unclear whether anxiety generally impairs attentional performance or if it may, at some point, lead to greater efficiency in the central area compared to peripheral visual regions.

Prior research has demonstrated that anxiety impairs the capacity of working memory (WM) (Eysenck & Calvo, 1992; Shackman et al., 2006). WM plays a pivotal role in regulating visual attention and executing current goals, responsible for holding templates for targets during perceptual selection and controlling actions (Oberauer, 2019), both in the physical or social environment (Stanković et al., 2019). Processes related to anxiety may compete with those related to the task execution due to WM interruption. Consequently, both negative valence (Plancher et al., 2019) and high arousal (Hou & Cai, 2022)—representing components of anxiety—may impact the WM of visual stimuli. This is a critical aspect related to maintaining attentional control.

Considering the inconsistent findings in existing literature regarding the influence of state anxiety on goal-directed attentional control, the present study used a dual-target paradigm to examine if state anxiety, when experimentally induced, would facilitate or hinder goal-directed attention. We aimed to understand the effects of such induced state anxiety on modulating goal-directed visual search over stimulus-driven attentional capture. In addition, by using both central and peripheral visual stimuli, we sought insights into whether state anxiety shapes spatial attention.

The current study focused on three key issues: (a) the impact of experimentally induced state anxiety on goal-directed visual search for dual targets by affecting top-down attentional control; (b) the effect of state anxiety on goal-directed visual search in an additional singleton search task; (c) the influence of state anxiety on goal-directed visual search under central and peripheral exposure, investigating the possibility of reduction/facilitation of spatial attentional narrowing. If state anxiety increases vigilance in general, increased state anxiety would enhance goal-directed attention control, as a brief exposure to a fearful movie is more likely to boost rather than impair attentional operations in WM. Furthermore, elevated state anxiety would lead to a more spatial narrowed attention, particularly noticeable when comparing central to peripheral visual search tasks.

## Method

### Participants

A group of 31 healthy university students (20 females), with a mean age of 26.06 years (SD = 3.99), recruited through a public announcement, participated in this study. They received either 9 Euro per hour or student credits for their participation. All had normal color perception and no neurological or psychiatric disorder. The study received approval from the Ethics Board of the Faculty of Psychology and Educational Sciences, Ludwig-Maximilians-Universität Munich, Germany, and all participants signed informed consent.

To determine the necessary sample size, we performed *Post hoc* power analysis using G*Power 3.1 (Faul et al., 2007). In prior study (Kim et al., 2021), the effect size was *d* = 0.61 for detecting effects between shock-treated and neutral groups. Our analysis used a two-tailed *t*-test to detect differences between two dependent means (matched pairs). With an effect size of *d* = 0.61 from a prior study, G*Power recommended a sample size of 31 participants for a power (1−ß) of 0.90, at an alpha level of .05.

### Apparatus and Stimuli

In the visual search task, participants were comfortably seated 70 cm in front of a monitor at eye level, using a chin-rest for keeping a stable distance. The search display consisted of either a central (radius of 4° of visual angle) or peripheral (radius of 6.9°) imaginary ring, with eight items spaced equidistantly around a fixation dot (0.2°  ×  0.2°). The central and peripheral search were equally likely. Each item (2.5°  ×  2.5° in diameter), either diamond or circle, contained either a vertical or a horizontal line (1.6°  ×  0.3°) inside, which was followed by a dot mask (7°  ×  7°). The eight items included two diamonds among six circles or two circles among six diamonds. The two pop-out shapes were target items, with its shape randomly swapped across trials. We alternated target shapes to promote dimension-based search over feature-based search, increasing the likelihood of stimulus-driven attentional capture by the distractor (Bacon & Egeth, 1994). Target and non-target items were green, while the distractor item was red, presented on 50% of all trials. All stimuli had a luminance of 14 cd/m^2^, set against a black background of 0.2 cd/m², measured by a Luminance and Color Meter (CS-100A).

To induce or reduce state anxiety, we followed the method of a previous study (Stanković & Nešić, 2020), using short movies with either anxiety-provoking or neutral content. The anxiety-inducing film was “The Shallows” (2016) (https://www.imdb.com/title/tt4052882/), featuring 6 min of tense and disturbing sequences, including a shark attack on a surfer, the buildup to the attack, psychological tension, and scenes of the surfer's shark-inflicted injuries. For the neutral condition, we used a 6-min segment from a National Geographic documentary on “The Great Wall of China” (https://www.youtube.com/user/NationalGeographic/videos), depicting the architecture of the Great Wall and people's relaxed daily lives. This contrast between neutral versus anxiety-inducing content helped control for random effects, such as participants’ mood or response biases. Stimuli were presented on a color-calibrated 24″ TFT-LCD monitor (ASUS VG248QE, screen resolution 1920  ×  1080 pixels, frame rate 120 Hz). Stimuli presentation and data collection were controlled by the self-coded PsychoPy (Peirce et al., 2019) program.

### Procedure

The experiment took place in a sound-attenuated and dimly lit cabin. Initially, participants filled out a state and trait anxiety form of the state and trait anxiety inventory (STAI), establishing a baseline. Next, they watched the first part (three minutes) of a movie, either with anxiety-inducing or neutral content, before starting the search task. Halfway through the trials, they watched another 3-min segment of the movie, followed by a second completion of the STAI inventory and the remaining trials. To measure changes in state anxiety, we subtracted the post-test from the pre-test STAI scores.

**Figure 1.**
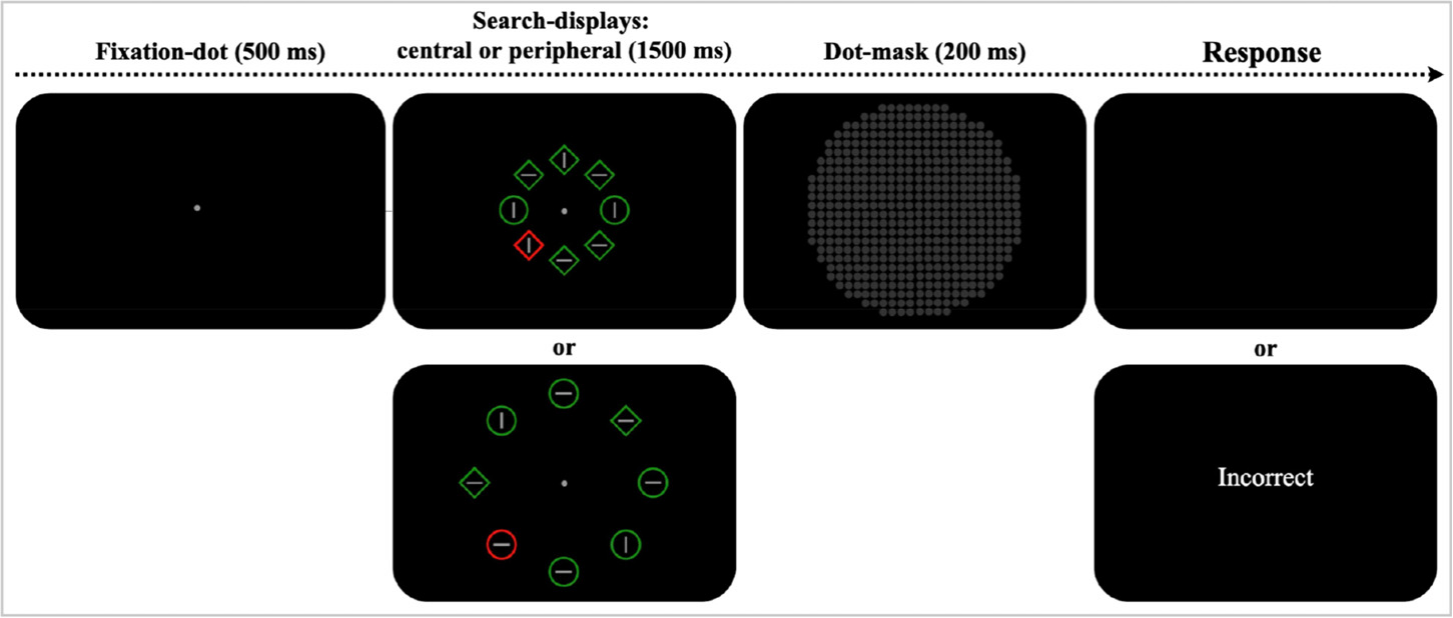
Illustration of the dual-target attention paradigm. *Note.* The visual search started with a fixation dot, followed by a search array with eight items. Among these, two were distinct shape targets (either two diamonds among six circles or vice versa), which were subsequently covered by a mask. An incorrect response was followed by text message feedback (“Incorrect”), while a correct response was followed by blank screen display. In this example, the upward central search display shows two target (i.e., a dual target) circles among homogeneous diamonds that pop-out/group in the visual search, whereas downward peripheral search-display shows two target diamonds among circles. Both the central and the peripheral presentation include a red item as a salient distractor, which occurred at 50% of the trials.

Each trial started with a fixation dot at the center of the screen for 500 ms, followed by a visual search display containing eight items, appeared for 1500 ms, and then immediately covered by a dot-mask for 200 ms (Figure 1). Participants were instructed to fixate on the dot and to search for two unique shape targets. Their task was to report the lines inside the two targets were “same” (both vertical or horizontal) by pressing the “J” key with their right-hand index finger or “different” (one vertical and one horizontal, or vice versa) by pressing the “F” key with their left-hand index finger, as fast and accurately as possible. The visual search display lasted up to 1500 ms or ended when a response was made. Incorrect responses triggered a 500-ms “Incorrect” message, whereas correct ones were followed by a 500 ms blank screen. The inter-trial interval lasted 500 ms. Participants’ reaction times (RTs) were measured from the onset of the search array to their button press.

To assess whether state anxiety influences goal-directed attentional control, we manipulated anxiety experimentally in a within-subject design. The study involved two separate sessions, spaced 7–10 days apart: the “anxiety session,” where state anxiety was induced by a short anxiety-provoking movie, and the “neutral session,” featuring a neutral movie to maintain participants’ calm emotional state. Two out of 31 participants reported they had seen the unpleasant movie prior to the experiment, while none were familiar with the neutral movie. We counterbalanced the order of the sessions across participants, randomly assigning them to begin with either the anxiety or neutral session.

Each session included 35 blocks, with 25 trials per block, followed by a short break. Before the main task, participants underwent 100 practice trials that mirrored the main task to get familiar with the task. These trials were excluded from statistical analysis. The potential sequential effects of practice trials were controlled for by employing a within-subject design.

Since the ACT posits that anxiety impairs efficiency (i.e., RT) more than effectiveness (i.e., accuracy) (Shi et al., 2019), we collected both RT and accuracy data. Efficiency reflects the ability to accomplish the task in the shortest time possible, while effectiveness refers to the success and accuracy of performance.

### Psychological Measurements

We assessed state anxiety using the STAI (Spielberger et al. 1983). The state anxiety section evaluates immediate feelings (e.g., “How you feel right now, that is, at this moment”) through 20 items (e.g., “I am worried,”), rated on a 4-point scale (from “Not at all” to “Very much so”). The trait-anxiety part, asking about general feelings (“…how you generally feel?), also contains 20 items (e.g., “I feel nervous and restless”), rated on a 4-point scale (from “Almost Never” to “Almost Always”). Participants reported their emotional states. Anxiety is characterized by a mixed subjective experience of anxious feelings, physical changes, and anxious thoughts (Stojiljković & Stanković, 2018). Normative scores ranged from 20 to 80, with higher scores indicating greater state anxiety. Internal consistency Alpha (*ɑ*), obtained during the first experimental session, was *ɑ* = .889 for state anxiety, and *ɑ* = .896 for trait anxiety. Alpha demonstrated a high reliability of used inventory.

### Data Analysis

Our experimental setup was within-participant repeated-measures design, spanning two sessions (anxiety-induced vs. neutral) to assess interplay of state anxiety and goal-directed attentional control. Practice session trials and RTs below 150 ms and above 3000 ms (on average, 3%) were excluded from statistical analysis.

We conducted a paired *t*-test to assess changes in subjective anxiety among participants, as indicated by the STAI inventory scores, during the anxiety and neutral sessions. We applied repeated-measures ANOVA on mean RTs and accuracies. Within-participant factors included session type (anxiety vs. neutral), distractor presence (present vs. absent), and target eccentricity (central vs. peripheral). We reported Cohen's partial eta-squared for effect size. Additionally, we calculated Bayes factors (BF_incl_) to further evaluate the likelihood of the alternative hypothesis.

Furthermore, we calculated distractor interference by the differences in RTs (ms) [distractor-present–distractor-absent] and accuracy (%) [distractor-absent–distractor-present presentation], as well as the eccentricity difference in RTs (ms) [central–peripheral] and accuracy (%) [central–peripheral]. Then we estimated intra-correlations using Pearson coefficient, examining relationships between subjective anxiety, distractor interference, eccentricity-related differences.

## Results

### Subjective Anxiety Measures

The mean subjective anxiety difference score, calculated as follows: [test-state minus pretest-state (i.e., the baseline) anxiety], indicated induced anxiety when the score was positive and a neutral/calm state and a reduction in anxiety when the score was negative. A paired *t*-test indicated a significantly higher subjective anxiety increase during the “anxiety” session (mean difference = 14.93, *SE* = 1.01) compared to the “neutral” session (mean difference = −2.45, *SE* = 0.54), *t*(30) = 6.52, *p* < .001, *d* = 1.17, BFincl > 100. This suggests that while the “neutral” session led to a slight decrease in anxiety, the “anxiety” session strongly increased it. In essence, we successfully manipulated the state anxiety in two sessions. The difference between anxiety and neutral sessions measured in baseline regarding trait anxiety was nonsignificant, *M* = 40.89, *SE* = 0.04, *M* = 40.91, *SE* = 0.04, *t*(30) = −0.153, *p* > .05.

### Visual Search Performance

On average, participants achieved 88.5% accuracy with a mean correct RT of 1333 ms. Although they were slightly faster during the anxiety session (*M* = 1321 ms, *SE* = 41 ms), compared to the neutral-sessions (*M* = 1344 ms, *SE* = 46 ms), this speed difference (23 ms) was not statistically significant, *t*(30) = −0.44, *p* = .662, *d* = −0.08, *BF* = 0.21. The accuracy rates between the anxiety (*M* = 88.4%, *SE* = 9%) and neutral sessions (*M* = 88.6%, *SE* = 11%) were also comparable, *t*(30) = −0.143, *p* = .887, *d* = −0.03, *BF* = 0.19.

**Figure 2.**
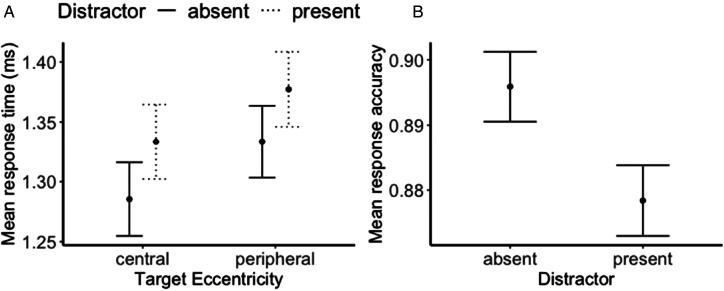
(A) Mean response time (RT) as a function of the target eccentricity, separated for the distractor presence (collapsed from two sessions, not significant). (B) Mean response accuracy as a function of the distractor presence (collapsed from the target eccentricity and sessions, both were not significant).

Regarding RTs, a repeated-measures ANOVA, featuring the main factors of Session Type, Distractor Presence, and Target Eccentricity, revealed RTs were comparable between two sessions, *F*(1, 30) = 0.195, *p* = .662, *BF*incl = 0.181. The Bayes factor favors the null hypothesis. However, both Distractor presence, *F*(1, 30) = 56.88, *p* < .001, $\eta_{p}^{2}$ = 0.65, BFincl > 100, and Target Eccentricity, *F*(1, 30) = 43.32, *p* < .001, $\eta_{p}^{2}$ = 0.59, BFincl > 100, were significant. Participants were quicker in trials without distractors compared to those with distractors. Similarly, responses were faster in the center relative to the peripheral presentations (a 50 ms central benefit) (Figure 2A). No interactions reached significance (*F*s < 1.96, *p* > .173, BFincls < 0.82).

For accuracy, the analysis similarly did not find significant effect of Session Type, *F*(1, 30) = 0.021, *p* = .887, $\eta_{p}^{2}$ < 0.001, BFincl = 0.04, or Target Eccentricity, *F*(1, 30) < 0.1, *p* = .969, $\eta_{p}^{2}$ < 0.001, BFincl = 0.13. Bayes factors favor the null hypothesis. However, the main factor of Distractor Presence was significant, *F*(1, 30) = 22.37, *p* < .001, $\;\eta_{p}^{2}$ = 0.43, BFincl = 97.74. Accuracy was higher in trials without distractors (89.6%) compared to those with distractors (87.8%) (Figure 2B). Additional, a significant interaction between Session Type and Target Eccentricity, *F*(1, 30) = 4.83, *p* = .036, $\;\eta_{p}^{2}$ = 0.01, was not confirmed in a post-hoc testing. No other interactions were significant (*F*s < 0.60, BFincl < 0.12).

Despite a significant difference in self-reported state anxiety, our main manipulation did not result in notable differences in mean RTs and accuracy. Consequently, we examined the individual relationship between state anxiety and both distractor interference and eccentricity benefits, as the group analysis between the neutral and anxiety session showed no significant correlation. Additionally, no correlation was found between subjective anxiety and distractor interference (absent-present trials) or target eccentricity effect (central-peripheral) (*rs* < 0.23, *p*s > .150).

## Discussion

In the present study, we investigated whether experimentally induced state anxiety affected goal-directed visual search. Specifically, we examined if elevated anxiety either enhances or hampers top-down attentional control in localizing and discriminating two targets. Additionally, we also investigated whether such anxiety influences bottom-up attention, potentially altering the extent to which distractor interference during visual search. Furthermore, we examined the role of state anxiety in either aiding or impairing the top-down attentional control.

Previous literature has yielded mixed evidence regarding whether anxiety reduces attentional effectiveness and efficiency or improves it, thereby reducing distractor interference. According to ACT, anxiety is seen as a key emotional factor that weakens top-down attention, favoring bottom-up attention (Derakshan & Eysenck, 2009; Eysenck & Derakshan, 2011). This suggests that reduced goal-directed attention might lead to increased distractor interference (Osinsky et al., 2012). A recent study (Kim et al., 2021) also reported that under threat conditions (such as electric shocks), individuals showed improved efficiency in goal-directed attention control, as the threat presence facilitated the visual search. Thus, participants experience less interference from task-irrelevant but physically salient distractor stimuli, suggesting enhanced top-down attention. This indicates that subjective anxiety might boost vigilance and alertness, reducing distractor interference. Such findings hint at biological survival mechanisms that activate in response to threatening stimuli (Ghassemzadeh et al., 2019; Vuilleumier, 2005). Increased internal state anxiety (arousal) enhances attentional vigilance, resulting in more effective goal-directed attentional control (Kim et al., 2021).

In our study, the experimental manipulation successfully heightened anxiety levels. However, this increased anxiety did not affect search performance in the dual-target attentional paradigm. When comparing groups across anxiety-session and neutral-session conditions, we found that experimentally indeed anxiety neither facilitated nor hindered goal-directed attention, as evidenced by similar RTs and accuracy in the dual-target visual search task. A possible interpretation of this null finding may be that state anxiety induced by the movie dissipated quickly, possibly due to modern movie viewers being somewhat immune to the unsettling aspects of movies.

Using goal-directed attention in the dual-target task may shift cognitive resources toward task demands, potentially lessening anxiety that is relevant to task performance (King & Schaefer, 2011). For example, in our experiment, the average RT for the dual-target task was 1330 ms. This contrasts with the average RT of below 600 ms in the classical additional singleton search task involving eight items and one target (Theeuwes, 1992), and 757 ms in a value-driven attention capture search task (Stanković et al., 2023). This suggests that the dual-target task we employed here is more challenging and participants become more vigilant. Recent studies (King & Schaefer, 2011; Vytal et al., 2012) have shown similar patterns, indicating that attentional performance is less affected when a demanding task occupies executive resources. High task demands redirect actions that might be influenced by anxiety towards achieving current goals. Such demands load the WM, which in turn prevents anxiety-related processes from influencing performance. Focusing on current goals thus diminishes potential anxiety impacts on visual search.

In contrast to Moser et al. (2012), who reported a correlation between the personality trait of anxiety and enhanced attentional distraction by physically salient stimuli in an additional singleton search task, our study did not support the alternative hypothesis. However, our experimental setup required a higher level of WM engagement due to its greater task demands, which may have activated attention compensatory mechanisms. Research suggests that anxiety might impair performance under low task loads but decreases when participants engage in a high-load task, as they refocus on current goals, occupying attention (Vytal et al., 2012).

Therefore, the lack of differences observed between sessions in our study might be due to compensatory mechanisms activated by the high demands of the task. ACT proposes that high anxiety levels may trigger compensatory mechanisms, which reduce distractor interference by employing additional cognitive resources (Eysenck & Derakshan, 2011). These additional resources are often mobilized through internal or external (e.g., reward) motivators. In our experiment, external feedback was provided via monetary compensation, independent of task performance. Though we cannot overlook this external motivator, compensatory mechanisms might also kick in as attention reallocates resources to cope with the WM load. In line with our findings is a neuroimaging study by Fales et al. (2008) which used neutral and negative videos to assess the influence of anxiety on cognitive efficiency in an n-back memory task. They reported no significant effects on both behavioral response accuracy and RT. Critically, the study did find a significant difference in neural network circuitry related to cognitive efficiency between low- and high-anxiety individuals. Thus, although Fales et al. (2008) observed no behavioral changes, their neuroimaging findings indicate that compensatory mechanisms are at play to mitigate anxiety's impact and maintain cognitive performance.

Furthermore, there is a hypothesis that suggests worry or apprehension, rather than arousal, is more susceptible to down-regulation through top-down control (Kalisch et al., 2006), since arousal typically primes mental capacities to respond to potential threats (Cornwell et al., 2011). We assumed that unpleasant movies primarily affected arousal rather than worry. Consequently, the absence of effects between sessions could imply that top-down control remains not overburdened in the dual-target setting.

The presence of the singleton distractor remained effective to impair search performance across all conditions. According to ACT, anxiety is thought to hinder the efficiency of suppression functions that resist interference from distractors and task-irrelevant stimuli (Eysenck & Derakshan, 2011), which could lead anxious participants to struggle in suppressing the salient singleton distractor due to diminished attentional control. However, the obtained failed to show any amplification of the distractor suppression. In that regard, the results demonstrated a stronger impact of bottom-up attention capture than the role of subjective anxiety in enhancing stimulus-driven inference.

Moreover, in line with expectation, both the anxiety and neutral sessions demonstrated more accurate responses in central compared to peripheral presentations, echoing recent findings in visuospatial detection task (Chandrakumar et al., 2020). Again, the robust central-preference effect was not anxiety-related, showing participants’ ability to maintain attentional performance despite elevated anxiety.

Admittedly, despite the observed increased subjective anxiety, the lack of impact of anxiety on attentional control might also partly stem from the type of videos we used. As we discussed earlier, modern participants might be less sensitive to the unsettling aspects of movies, in comparison with those highly aversive stimuli such as electric shock. This limits our ability to draw firm conclusions about the impact of anxiety on goal-directed attentional control. Further research could consider exploring whether increasing the intensity of anxiety to more aversive levels might impair goal-directed attentional control, as suggested by the ACT.

To conclude, our study, featuring a unique dual-target task combined with the manipulation of state anxiety through video viewing, confirmed the attentional capture and central-preference effects. However, despite strong differences in subjective state anxiety across sessions, this state anxiety did not meaningfully impact search performance. We suggest that this resilience might stem from compensatory mechanisms that offset anxiety, a result of the high demands and WM load inherent in the dual-target visual search task. To validate these compensatory mechanisms, further investigations using electrophysiology or brain imagining are necessary.
